# *Cynara cardunculus* as a potential source of milk coagulating protease: Effects on physical properties of cow's milk

**DOI:** 10.1002/fsn3.2981

**Published:** 2022-07-18

**Authors:** Abir Mokni Ghribi, Ines Makhlouf Gafsi, Christophe Blecker, Hamadi Attia, Mohamed Ali Bouaziz, Souhail Besbes

**Affiliations:** ^1^ Ecole Nationale d'Ingénieurs de Sfax, Laboratoire Analyse Université de Sfax Sfax Tunisie; ^2^ Gembloux Agro Bio‐Tech, Unité de Technologie des Industries Agro‐Alimentaires, passage des Déportés 2 Université de Liège Gembloux Belgium

**Keywords:** coagulation, *Cynara* extract, milk, rheology, stability, texture, wild cardoon

## Abstract

In the present research study, *Cynara cardunculus* (wild cardoon) flowers were blended and extracted using different types of buffers (phosphate buffer; citrate buffer and distilled water) for different maceration times. The most reliable, quick and efficient buffer was found to be phosphate (pH = 6.5) with a 6‐h maceration time, which was used throughout this study. *C. cardunculus* extract (CE) was found to have high clotting and proteolytic activities. The extracted enzyme was found to be very stable against a wide range of pH values as well as of temperature. The formation of milk gels prepared in the presence of CE with different types of milk was evaluated using dynamic rheology and Turbiscan. The evolution of both elastic (G′) and viscous (G″) moduli was monitored with time. The values of the whole milk enriched with milk powder gels were higher. Coagulum stability was evaluated using Turbiscan. The textural properties and the curd‐firming rate of coagulum were also determined. In conclusion, the prepared CE could be an efficient milk‐clotting agent in the production of dairy products.

## Graphical abstract

‐Cynara cardunculus flowers were extracted using different types of buffers for different maceration times ‐Formation of milk gels prepared in the presence of CE with different types of milk was evaluated using dynamic rheology and Turbiscan ‐ Textural properties and the curd‐firming rate of coagulum were determined

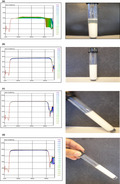

## 1 INTRODUCTION

The global cheese market is valued at ~$100 bn, and global cheese consumption is expected to increase by ~13.8% between 2019 and 2029 (OECD/FAO, 2020). According to Salque et al. (2013), cheese has been part of the European diet for 6000–8000 years. Now, it is gaining popularity in countries where it was not traditionally part of the national diet, likely due to the Westernization of the diet (OECD/FAO, 2020). Cheese making was all art, but the development of technology and science was necessary to make progress in producing a higher quality cheese and to look for other rennet substitutes. Traditional cheese making could not keep up with the demand for cheese, and the advancement of the factory system was necessary. Moreover, in view of this large world production and consumption of cheese and the world shortage of animal rennet, several research projects have been launched for several years on coagulating enzymes likely to be used in cheese making. The industry should be now able to produce, in practically unlimited quantities, these substitutes at competitive prices. For this reason, this work focuses on the extraction of proteases from *Cynara cardunculus* to use them in milk coagulation.

Cheeses, very popular food produced worldwide, are generally nutrient‐dense foods and are a valuable source of high‐quality proteins, lipids, vitamins (e.g., vitamin A, B2 and B12) and minerals (particularly calcium and phosphorus). It is well documented that cheese provides all essential amino acids except methionine and cysteine in more than the recommended quantities for children or adults (Tomé et al., 2002). In addition to macro and micronutrients, some matured cheeses contain bioactive components (e.g., bioactive peptides), which have health benefits, while beneficial bacteria present in the cheese matrix can potentially improve human gut health by producing short‐chain fatty acids (FA) (Santiago‐López et al., 2018). Moreover, some FA found in milk, including butyric acid (4:0), conjugated linoleic acid (CLA) as well as branched‐chain FA, have been reported to have positive health outcomes, such as maintenance of gut microbiota, weight control, gut health at birth and the prevention of chronic inflammatory diseases (Bruen et al., 2017; Gómez‐Cortés et al., 2018). Cheese consumption was correlated with beneficial metabolic health markers. In addition, according to different studies, an anti‐cariogenic, an anti‐obese, an anti‐carcinogenic and an anti‐hypertensive effect were reported for cheese product consumption (Feeney et al., 2021; Verruck et al., 2019; Walther et al., 2008).

Milk coagulation, a crucial step in most cheese‐making processes, is caused by the action of proteases on milk proteins. The specific hydrolysis of the peptide bond Phe105‐Met106 in ĸ‐casein provokes casein micelle destabilization and subsequent aggregation, resulting in the transformation of the milk into curd. Different types of proteases have been used for this purpose, with natural, microbial and recombinant sources being the most common (Kumar et al., 2010). However, the high price of rennet and limited supply diet (vegetarianism), religious factors (e.g., Judaism and Islam) and ban on recombinant calf rennet (in France, Germany and the Netherlands) have pushed researchers to search for other sources (Shabani et al., 2018). As an alternative, several proteases obtained from vegetal sources such as fruits (e.g., kiwi, melon and papaya), roots (e.g., ginger rhizome), latex (e.g., Papaya fruit Carica p. and Sodom apple *Calotropis procera*) and flowers (e.g., *C. cardunculus* and *Centaurea calcitrapa*) have been recommended for use as milk clotting agents (Adetunji & Salawu, 2008; Domsalla & Melzig, 2008; Fernandez‐Salguero et al., 2002). Additionally, the functional properties of proteases from plant extracts are critical to define the unique texture and flavor of these products. Actually, these properties may open the path for other innovative applications of these proteases.

*Cynara cardunculus*, a plant source, has been used for ages as milk coagulant (Fernandez‐Salguero et al., 2002). Many researchers have shown that it is possible to extract and purify two aspartic proteases, namely cardosins A and B, from the flowers of the thistle. Each cardosin consists of two subunits, with apparent molecular weights of 31 kDa and 15 kDa for cardosin A, and 34 kDa and 14 kDa for cardosin B (Veríssimo et al., 1995, 1996).

To the best of our knowledge, few studies have been devoted to the investigation of the effect of pH buffer and temperature on enzymatic activities of wild cardoon rennet (Ben Amira, Mokni et al., 2017). Thus, it is interesting to evaluate the milk coagulating activity (MCA) and the proteolytic activity (PA) at different rennet pH values. Although the evaluation of MCA and PA is crucial for the selection of the best calf rennet substitute, the determination of curd quality is not of less importance, and mainly implies physico‐chemical characteristics and texture analysis, which are included in sensory perception.

The choice of milk type is also considered as an essential factor in the gelation step of cheese making. It can influence the aggregation reactions and colloidal stability of coagulum. It is so important to study the visco‐elastic characteristics of milk gels produced by *C. cardunculus* coagulants, prepared with different milk types. The gels' formation was monitored using dynamic rheology. Turbiscan was used in this study to characterize suspension instability.

Therefore, the objective of the present study is firstly to evaluate the potential use of *C. cardunculus* flowers as a new source of milk‐clotting proteases by studying the effect of pH extraction on physico‐chemical properties and enzymatic activities of crude extracts. The effect of temperature on MCA and PA of crude CE was also evaluated. As for the second objective, it is to monitor gel formation and compare the gelation properties of curd produced by these coagulants, using dynamic rheology and a Turbiscan instrument. A comparison was made with different types of milk, in an attempt to reveal interactions and better understand what happens when such a coagulant is used in cheese making process. This study allows the investigation of the technological applicability of *C. cardunculus* rennet prepared and selects the best enzyme preparation and conditions, revealing the optimal properties for cheese production.

## 2 MATERIALS AND METHODS

### 2.1 Plant material

The harvesting of the *cynara* flowers for vegetative flowering stage to take place in the period ranging from the end of March and beginning of April. In the vegetative stage, flowers are still closed. All collected samples were then stored immediately at 20°C.

### 2.2 Preparation of the *cynara* flower powder

The flowers are ground using a mechanical mill (Retsch Grindomix GM 200) for 20 s at a speed of 7000 rpm to obtain a fine flour (1–2 mm diameter). The powder obtained is then fractionated and stored in glass vials at 4°C.

### 2.3 Extract preparation

The crude extracts of wild cardoon flowers were prepared by the methods described by Ben Amira, Mokni et al. (2017) with some modifications. The crude enzyme extract was prepared by macerating a ground material of *cynara* in the solvent of extraction (distilled water pH = 7; phosphate buffer pH = 6.5; citrate buffer pH = 5.9) for different maceration times (2–6 h) at 25°C in a water bath (GFL 1083) and with gentle agitation. The aqueous extract thus obtained was centrifuged for 5 min at 6000 *g* (Jouan C312). The extract was stored at 4°C and either used on the same day for enzymatic activities or frozen at −20°C until further use.

#### 2.3.1 Determination of coagulating activity

The clotting activity was determined according to the procedure described by Berridge (1952). One rennet unit (RU) was defined as the amount of crude extract needed to coagulate 10 ml of reconstituted low heat skim milk powder (NILACTM, NIZO) at 30°C in 100 s (Berridge, 1952). The assays were carried out in triplicate and their averages were used as datum points.

#### 2.3.2 Determination of proteolytic activity

The PA was determined according to the methods of Kunitz (1947) with slight modifications. It involves the use of bovine casein (casein for biochemistry, Merck KGaA, 64,271) as a substrate. A volume of 2% casein solution in 0.1 M phosphate–citrate buffer (pH 6.5) was incubated at 30°C for 5 min. The reaction was initiated by adding the coagulant extract or calf chymosin (Berthelot 530) and the mixture was incubated for 4 h at 30°C. The reaction was then stopped by the addition of cold 5% (w/v) trichloroacetic acid (TCA). Next, the mixture was vortexed, left to stand for 15 min at 30°C, and then centrifuged at 14,900 *g* for 10 min. The enzymatic activity is based on the amount of peptide released in the TCA. The absorbance of the resulting solutions was measured spectrophotometrically at 280 nm (UV mini 1240, UV/VIS spectrophotometer, SHIMDZU).

#### 2.3.3 Determination of protein content

The protein content was determined according to Bradford method using Bovine Serum Albumin as standard (Bradford, 1976).

A volume of 0.1 ml of *cynara* extract was mixed with 3 ml of Bradford protein assay. The blank was conducted in the same manner except that distilled water was used instead of the sample. Thereafter, the absorbance was measured at 595 nm.

### 2.4 Study of optimal coagulation conditions

#### 2.4.1 Effect of optimal milk temperature

The influence of the incubation temperature on the milk clotting activity of CE was determined in a temperature range of 25–75°C by setting the temperature to the following values: 25; 30; 35; 40; 45; 50; 60; 70; 75°C. The milk was incubated in a water bath (1004 GFL), and the temperature of the milk was measured by a thermometer. The MCA was determined for each temperature value and given by the average of three replicates.

#### 2.4.2 Effect of optimal pH milk

The effect of the pH on the coagulation activity of *cynara* was studied. The pH of the milk was adjusted to the following values: 5.3; 5.8; 6.3 and 6.8. The measurement of pH of the milk was made using pH meter using an MP 220 pH meter (Mettler‐Toledo GmbH). The measurement was carried out by introducing the two sensors (pH and temperature) in a milk sample preheated to a temperature of 20–25°C. The clotting activity was measured for each pH value.

### 2.5 Characterization of the obtained coagulum

Four types of milk were selected for this part of study: whole milk (WM), skimmed milk (SM), as well as whole milk enriched with milk powder (5%) and (10%) (WME 5%; WME 10%).

#### 2.5.1 Evaluation of the stability of the coagulum emulsion

The stability of the obtained coagulum emulsion was evaluated by the Turbiscan MA 2000 (Formulaction) (Mengual et al., 1999). The apparatus, equipped with a near‐infrared light source (880 nm), moves up and down along a flat‐bottomed cylindrical cell. The light source scans the sample, at 1 h intervals, from top to bottom and measures the percentage of backscattered or transmitted light during 3 h at 30°C incubation. The variation in the levels of backscattering is associated with changes in the concentration of the rennet‐induced coagulation and particle size.

#### 2.5.2 Texture profile analysis

The texture profile coagulum was characterized according to the procedure TPA (Texture Profile Analysis) by applying two compression cycles using a texture analyzer TA‐XT2 SMS fitted with a cylindrical probe plexiglass (2 cm in diameter × 2 cm in height). The cylindrical curds were cut using a plastic box of 13 mm high and ultra‐thin fishing line to get curds with dimensions of 4.5 cm diameter and 13 mm height. These curds were held 10 min at room temperature, before evaluation. The samples were analyzed at 20°C. The compression was applied over a distance of 0.004 m, with the probe moving at the speed of 0.002 m/s, a pre‐speed of 0.005 m/s, post a speed of 2 mm/s and a rest period between two cuts of 20 s (Ben Amira et al., 2018).

#### 2.5.3 Rheological properties

Before comparing visco‐elastic properties of gels, the enzyme amount added to different type of milk was standardized in order to obtain a visual gel produced by the optimized *C. cardunculus* extract. Then, different types of milk (10% prepared in 0.01 M CaCl_2_ at pH 6.5), were firstly preheated at 30°C for 15 min. After that, *C. cardunculus* extract was added to 10 ml of milk samples and milk gelation was monitored during 30 min at 30°C.

The rheological properties of the renneted‐milk samples were evaluated at 30°C for 2 h using an MCR 302 Rheometer (Anton Paar) with an oscillatory mode. The measuring geometry used was a Cup and Bob (50 mm, 1°). The employed oscillation frequency was fixed at 0.1 Hz and the rheometer was programmed automatically to give a strain of 0.03%, which was found to be within the linear visco‐elastic region of rennet milk gels (Zoon et al., 1988).

Gelation time (GT) is defined as the time when aggregated gel presents a G' of 1 Pa, as reported previously by Zoon et al. (1988) and Klandar et al. (2007).

### 2.6 Statistical analysis

The analytical values were taken using three independent determinations. The obtained results were expressed as mean values ± standard error of three independent determinations. As for the statistical analyses, they were conducted using a statistical software program (SPSS for Windows version 20.0). The data were subjected to the analysis of variance using the general linear model to determine significant differences between the samples (*p < .05*).

## 3 RESULTS AND DISCUSSION

### 3.1 Optimization of extraction conditions

#### 3.1.1 Influence of pH and incubation time on protease extraction

**FIGURE 1:**
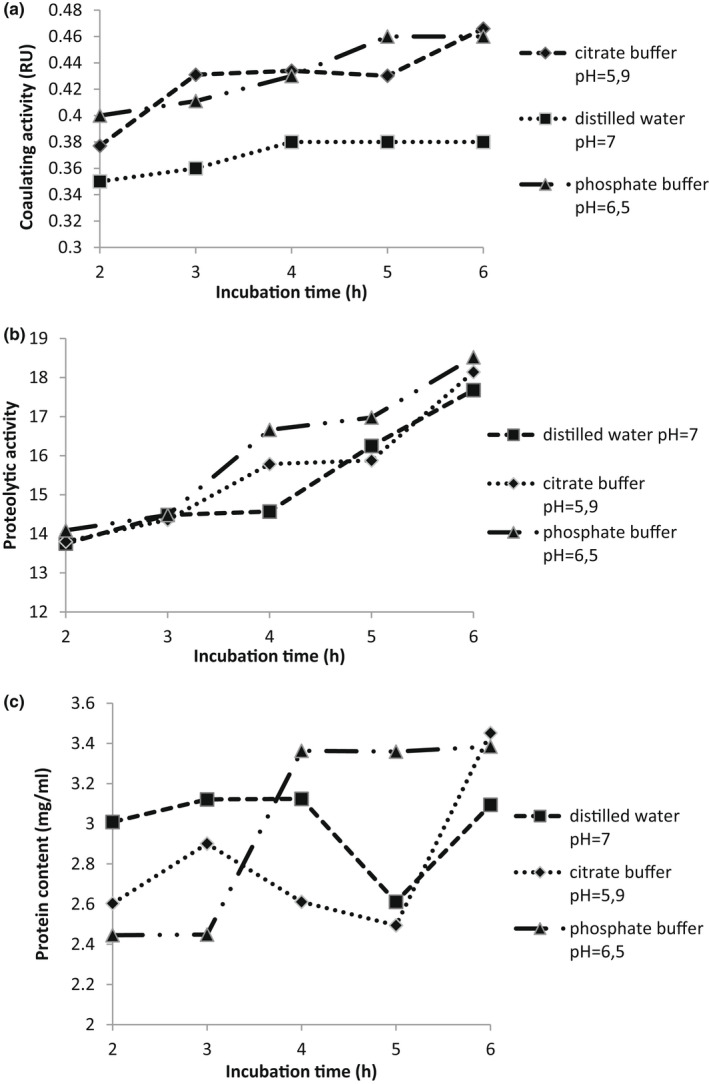
Evolution, with incubation time, of the coagulating activity (a), the proteolytic activity (b) and protein content (c) of crude extract at pH = 7(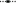); pH = 6.5 (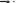) and pH = 5.9 (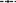)

The evolution of the coagulating, the PA and the protein content of crude extract from *Cynara* incubated at different pH values and incubation times is plotted in Figure 1. The change in the clotting activity of the crude extract according to the maceration time shows that the maximum activity was recorded after 4 and 5 h for the phosphate and citrate buffer. For the distilled water, we note that activity increases to reach its maximum after 4 h, but remains lower than that of citrate and phosphate buffer.

Plant tissues contain a wide range of proteins that vary substantially in their functions and properties. The protein content of prepared *Cynara* flowers extract (CFE) was in the range of 2.4–3.4 mg/ml. It was reported that protein content for the lyophilized crude extract from the flowers of *C. cardunculus* was 15–18 g/100 g of the total protein (Fernandez‐Salguero et al., 2002). Many factors such as manufacturing conditions and composition of the enzyme preparation have a considerable impact on protein content. Overall, the protein content of *Cynara* extract increases with the maceration time flowers in the extraction solvent. After 6 h of soaking, it is clearly remarkable that proteases extracted in phosphate buffer have the highest protein content.

Therefore, phosphate buffer was used as an extracting buffer throughout the study with a 6 h maceration time.

### 3.2 Temperature and pH effect on milk‐clotting activity of *cynara* flower extracts

**FIGURE 2:**
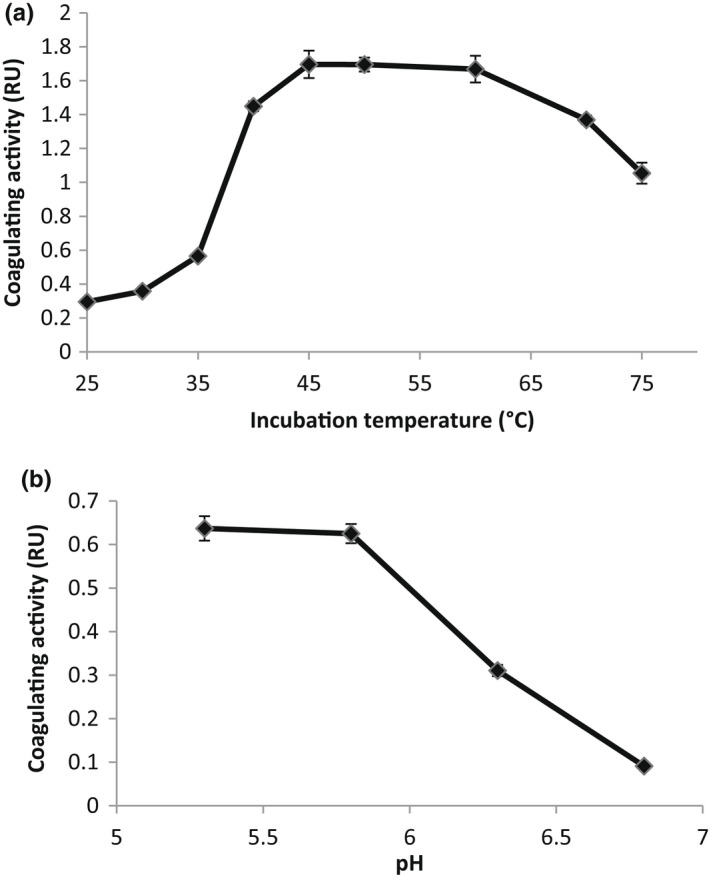
Temperature (a) and pH (b) effect on milk‐clotting activity of crude extract

The effect of the temperature on the milk‐clotting activity of CFE is shown in Figure 2a. The results of this study indicated that coagulating activity increased with temperature in a range of 25–45°C, reaching a maximum between 45 and 60°C. This maximum corresponds to a value of 1.668 RU, while at 25°C, this activity was 0.295 RU. Temperatures higher than 60°C decreased the coagulating activity, possibly due to the enzyme denaturation (Figure 2). Such a high thermal stability shows an excellent scope of the *cynara* enzyme for use in dairy industry.

The optimal temperature was also found to be 60°C for capparin from *Capparis spinosa* (Demir et al., 2008), protease from *Ginger rhizome* (Hashim et al., 2011), *Synadenium grantii* enzymes (Menon et al., 2002), and protease from *Euphorbia amygdaloides* (Demir et al., 2005). Procerain from *Calotropis procera* was optimal in the temperature range of 55–60°C (Dubey & Jagannadham, 2003).

The effect of the pH on the coagulating activity of CFE is shown in Figure 2b, revealing that the activity decreases with pH in the range of 6–7. A peak of maximum activity was found at acidic pH (around pH 5.0), which could indicate the presence of acid proteases. The peak specific activity of the *cynara* protease at pH 5.5 was in agreement with the pH optima of other plant cysteine proteases (Demir et al., 2008; Yamada et al., 2000). For acid pH values, 75–90% of the extracted enzymes correspond to cardosin A (Cavalli et al., 2013). Ben Amira, Mokni et al. (2017) reported that the extraction pH was the most influential factor on MCA, due to its significant impact on the level of extracted cardosin A. This enzyme was shown to be similar to chymosin, in terms of specificity and kinetic parameters, by cleaving the same peptide bond (Phe105–Met106) of the casein (Cavalli et al., 2013). On the other hand, increasing pH buffer on rennet activity could be explained by the fact that, at a high pH value, the extraction of non‐proteolytic enzymes, is more pronounced. These compounds interfere with enzyme tests and may promote the development of unnecessary reactions, thus causing under‐estimation of the activity. Furthermore, the high content of phenolic compounds involves their swift oxidation to form pigments, which may attach to native enzymes, thus leading to their inactivation (Barros et al., 2001). All commercially available rennets exhibit a curvi‐linear increase in relative milk clotting activity as pH drops from pH 7 towards pH 4–5 (Harboe & Budtz, 1999). According to Kuchroo and Fox (1982), the release of water‐soluble nitrogen (WSN) in cheese is primarily a result of casein solubilization caused by the action of proteolytic enzymes, and it is traditionally referred to as a measure of “ripening index”. Protein hydrolysis in the cardoon‐coagulated cheese was more extensive, leading to the formation of a separate group of samples with high “ripening index”.

### 3.3 Coagulum stability

Turbiscan was used in this paper to characterize aggregates migration during milk gelation by *C. cardunculus* extract prepared with different milk types.

**FIGURE 3:**
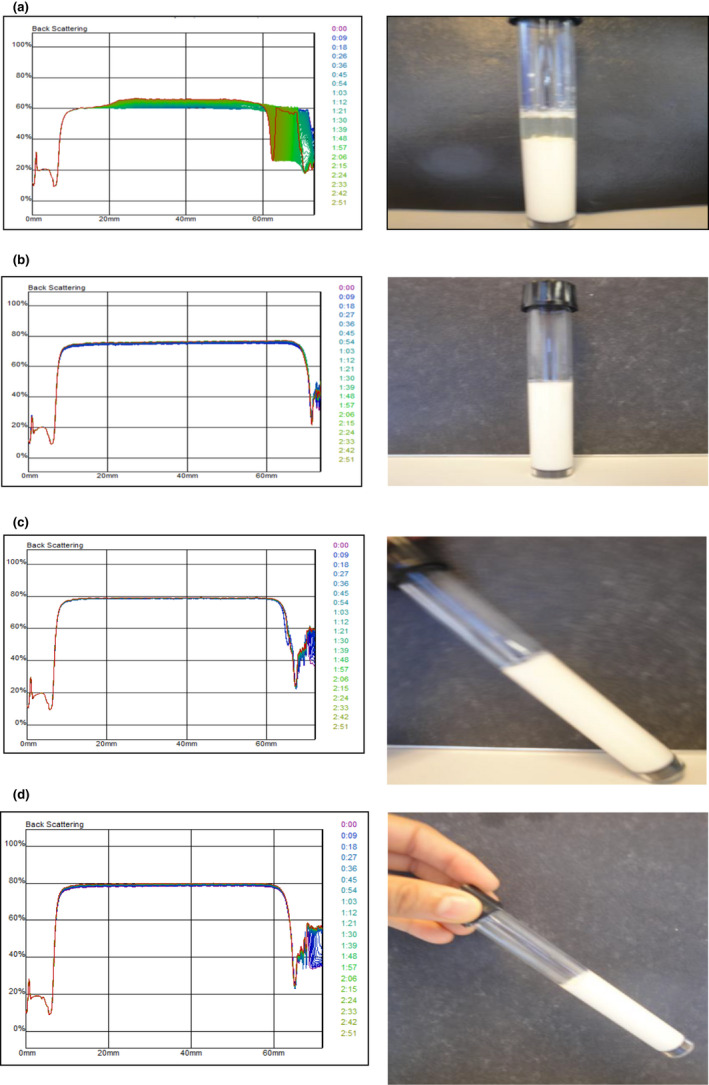
Backscattering profiles of skimmed milk (a), whole milk (b), whole milk enriched with milk powder (5%) (c) and whole milk enriched with milk powder(10%) (d) coagulated with cynarase flower extract

As shown in Figure 3, the crude extract greatly coagulated the whole milk and whole milk enriched with milk powder (5% and 10%) compared to skimmed milk sample.

Milk coagulation occurred when raw CFE was used as the coagulant, indicating that *cynara* flower can be a good source of clotting enzymes. The evolution of delta back scattering (∆BS) throughout the coagulation process of WM and WME (5% and 10%) and SM sample was investigated and presented in Figure 3. It indicated that during milk coagulation, a continued increase in the ∆BS was observed after 1 and 2 h, for WM and WME (5% and 10%), revealing positive values. Profiles remained almost constant during rennet‐induced coagulation, suggesting that the coagulation process takes place throughout the whole tube and induced only a constant change of the system, which is related to the particle size aggregation or variation. Hence, the increase in the back scattered light revealed a protein aggregation that scattered the light more intensely (Zhao et al., 2014).

The changes in ∆BS were more organized for gel produced by WM than for that prepared with SM, reflecting a more uniform texture of gel produced by *C. cardunculus*. Particles migration occurred for skimmed milk, but appeared instead the coagulation of particle clusters once.

### 3.4 Rheological properties

Monitoring rheological properties is one of the means to measure gel formation during cheese manufacturing. It is also a powerful tool to examine the impact of enzymatic hydrolysis of caseins and evaluate enzyme specificity changes on the functional properties of milk proteins (Esteves et al., 2003). In the present study, dynamic rheology was employed to evaluate gelation properties and compare the visco‐elastic characteristics of gels produced by *C. cardunculus*, with different milk types. The obtained results have shown that the gelation curves produced by *C. cardunculus* coagulants were similar, due to the similar action of these enzymes on milk casein. The mechanical properties of the milk curd are related to network composition, structure, and interactions among molecules within the network (Lucey et al., 2003).

**FIGURE 4:**
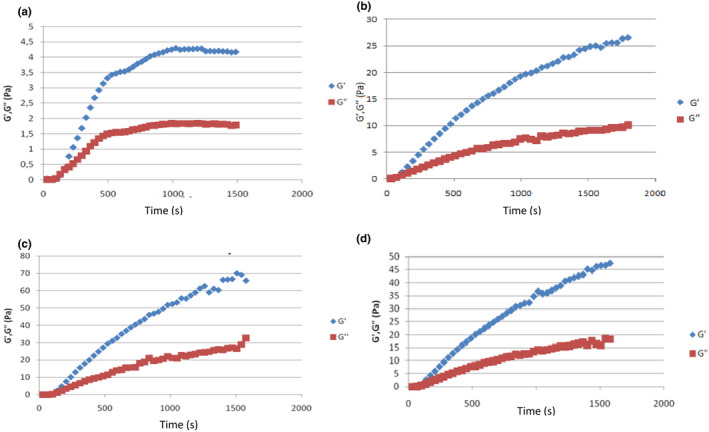
Evolution of G′and G″ versus time during coagulation of skimmed milk (a), whole milk (b), whole milk enriched with milk powder (5%) (c) and whole milk enriched with milk powder(10%) (d)

The evolution of the complex modulus (G) with time is shown in Figure 4, and a sigmoidal increase in G with time was observed. Shabani et al. (2018) reported that G′ value was higher than that of G′′ at the outset and throughout the experiment for ficin and *Polyporus badius* extract, revealed the association of proteins. Bohlin et al. (1984) pointed out that this is the typical behavior of the secondary phase of milk coagulation. Although the G values increased over time, a plateau was not reached in the case of whole or enriched milk. In agreement with the results reported by Silva et al. (2003), a sigmoidal increase in G* with time for cardosin A and cardosin B from *C. cardunculus* was observed. This increase in this modulus can be explained by the reduction of electrostatic repulsion between micelles, which may lead to faster structural rearrangements, and subsequent micelle aggregation. In general, cheeses made using cardoon were found to be more hydrolysed than those made using animal rennet. Similarly, those made with regular milk were more hydrolysed than those made using concentrated milk (Agboola et al., 2009).

The highest value of G* (i.e. 70 Pa) was obtained for whole milk enriched with milk powder (10%), whereas the lowest (i.e. 4.3 Pa) was obtained for skimmed milk.

### 3.5 Textural properties

Table 1 shows the textural changes in TPA profile of all samples (SM, WM, WME (5% and 10%)). This result is probably due to the initial quality of milk. Coagulum made using WME was harder and more adhesive than those made using SM and WM. This difference can be explained by the difference in the total solid concentration. Although there has not been evidence yet in published literature, it is possible that some casein micelles may be physically damaged when milk powder was added. This may in turn affect the coagulation process when making cheese, resulting in a hard and crumbly product. Low et al. (2006) reported that the use of cardoon extract led to enhanced lipolysis. In addition, lipolytic activity in other proteases such as those used in the commercial microbial coagulant has been reported (Somkuti & Babel, 1968, Agboola et al., 2009).

**TABLE 1:** Textural parameters of different milks: Skimmed milk, whole milk, whole milk enriched with milk powder (5%) and whole milk enriched with milk powder (10%)

	Skimmed milk	Whole milk	Whole milk enriched with milk powder (5%)	Whole milk enriched with milk powder (10%)
Hardness (N)	0.130 ± 0,034^a^	0.128 ± 0,005^a^	0.369 ± 0,025^b^	0.770 ± 0,046^c^
Elasticity (mm)	0.762 ± 0,065^a^	0.912 ± 0,046^b^	0.946 ± 0,026^b^	0.971 ± 0,021^b^
Cohesivness	0.450 ± 0,053^a^	0.360 ± 0,066^b^	0.332 ± 0,010^b^	0.350 ± 0,002^b^
Adhesivity (J)	−0.118 ± 0,008^a^	−0.163 ± 0,033^b^	−0.284 ± 0,004^c^	−0.319 ± 0,009^d^
Gumminess (N)	0.104 ± 0,075^a^	0.063 ± 0,023^a^	0.129 ± 0,009^a^	0.284 ± 0,007^b^
Chewiness (J)	0.087 ± 0,073^a^	0.080 ± 0,025^a^	0.117 ± 0,006^a^	0.285 ± 0,004^b^

^a–d^ Different letters in the same line indicate significant differences (*p* ≤ .05).

The difference in the extent and pattern of hydrolysis results in the changes in the coagulation properties of skimmed milk compared with whole milk with the same protein concentration. The absence of fat in the skim milk leads to a limited lipolytic activity, thus forming a coagulum having characteristics that are significantly different from those of the whole milk.

### 3.6 Curd firming rates

**FIGURE 5:**
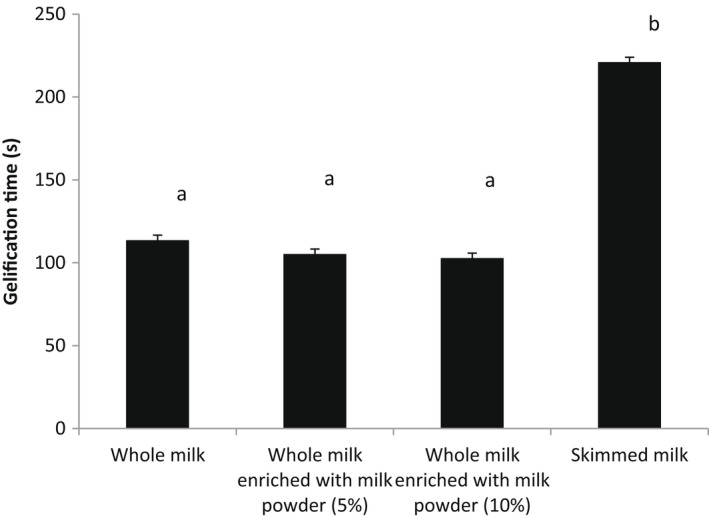
Curd firming rates for different milks: Skimmed milk, whole milk, whole milk enriched with milk powder (5%) and whole milk enriched with milk powder(10%)

The gelling point (Tgel) is defined as the time when an infinite network occurs in the sample. The curd firming rates for the different skimmed milk, whole milk, whole milk enriched with milk powder 5% and 10% is depicted in Figure 5. The curd‐firming rate for skimmed milk was higher than those for whole milk and whole milk enriched with milk powder. The absence of fat may be responsible for this observation. Shabani et al. (2018) reported that the concentration of enzymes was profoundly affected the gelation of milk protein. In addition, the milk proteins need more time to aggregate at lower concentrations of mushroom enzymes. Based on informations from previous studies, GT was influenced considerably by the increase of the *C. cardunculus* amount found by Ben Amira, Mokni et al. (2017). A significant decrease (*p* < .05) of about 55% was recorded for raw skim milk and 54% for reconstituted skim milk (Ben Amira, Mokni et al., 2017). Indeed, the addition of crude extract from flowers, containing polyphenols, colored pigments, proteins and different types of aspartic proteases (cardosins G, H, E and A) (Ben Amira et al., 2016, 2017) may contribute to the slow action of cardosin A, towards ĸ‐casein peptide bond, at the beginning of gelation.

## 4 CONCLUSION

With the advancement of science and technology, the use of various traditional methods may decrease. This study indicated the effect of the *cynara* extract on the processes of milk coagulation. Proteolytic, textural and rheological studies showed significant differences between different types of milk (skimmed milk, whole milk, whole milk enriched with milk powder [5% and 10%]). The study also showed that the superior properties of coagulum from concentrated milks, when coagulated with cardoon, may not be merely a related concentration. They are also probably due to the intrinsic properties of its casein micelles, e.g., proportion and size of individual casein molecules, which were left mostly undisturbed. Further studies on the dynamic rheological and microstructural properties of the milk should help in elucidating the molecular changes that underpin the gross structural observations reported in this study. The application of the cardoon extract to coagulate milk for the manufacture of commodity‐type cheeses is currently being investigated. The acceptability of cheese to the final consumer largely depends on specific sensory characteristics, including flavor and aroma. In fact, the relationship between the sensory attributes of products and consumer preferences has been identified as one of the main pillars to increase the chance of success in the market. Additional sensory studies based in consumer perception and acceptance test with consumers should be added.

## CONFLICTS OF INTEREST

The authors declare that there is no conflict of interests regarding the publication of this paper.

## Data Availability

The Data that support the findings of this study are available from corresponding auther “Abir MOKNI” upon reasonable request.
